# Drp1-mediated mitochondrial fission promotes renal fibroblast activation and fibrogenesis

**DOI:** 10.1038/s41419-019-2218-5

**Published:** 2020-01-16

**Authors:** Yating Wang, Miaoqing Lu, Liping Xiong, Jinjin Fan, Yi Zhou, Huiyan Li, Xuan Peng, Zhong Zhong, Yihan Wang, Fengxian Huang, Wei Chen, Xueqing Yu, Haiping Mao

**Affiliations:** 1 Department of Nephrology, the First Affiliated Hospital, Sun Yat-sen University, Guangzhou, China; 2 NHC Key Laboratory of Nephrology, Guangzhou, China; 3 Guangdong Provincial Key Laboratory of Nephrology, Guangzhou, China; 4 Department of Pathology, the First Affiliated Hospital, Sun Yat-sen University, Guangzhou, China; 5 Laboratory for Kidney Pathology, Inc., Nashville, TN USA

**Keywords:** Energy metabolism, End-stage renal disease

## Abstract

Excessive mitochondrial fission acts as a pro-proliferative marker in some cancers and organ fibrosis; its potential role in renal fibroblast activation and fibrogenesis has never been investigated. Here, we showed more pronounced fragmented mitochondria in fibrotic than in non-fibrotic renal fibroblast in humans and mice. In a mouse model of obstructive nephropathy, phosphorylation of Drp1 at serine 616 (p-Drp1S616) and acetylation of H3K27(H3K27ac) was increased in fibrotic kidneys; pharmacological inhibition of mitochondrial fission by mdivi-1 substantially reduced H3K27ac levels, fibroblasts accumulation, and interstitial fibrosis. Moreover, mdivi-1 treatment was able to attenuate the established renal fibrosis. In cultured renal interstitial fibroblasts, targeting Drp1 using pharmacological inhibitor or siRNA suppressed TGF-β1-elicited cell activation and proliferation, as evidenced by inhibiting expression of α-smooth muscle actin (α-SMA) and collagen I, as well as by reducing DNA synthesis. In contrast, Drp1 deletion enhanced cell apoptosis, along with decreased mitochondrial fragmentation, mtROS elevation, and glycolytic shift upon TGF-β1 stimulation. In Drp1 deletion fibroblasts, re-expression of wild-type Drp1 rather than Drp1S616A mutant restores the reduction of TGF-β-induced-Drp1 phosphorylation, H3K27ac, and cell activation. Moreover, TGF-β1 treatment increased the enrichment of H3K27ac at the promoters of α-SMA and PCNA, which was reversed in Drp1-knockdown fibroblasts co-transfected with empty vector or Drp1S616A, but not wild-type Drp1. Collectively, our results imply that inhibiting p-Drp1S616-mediated mitochondrial fission attenuates fibroblast activation and proliferation in renal fibrosis through epigenetic regulation of fibrosis-related genes transcription and may serve as a therapeutic target for retarding progression of chronic kidney disease.

## Introduction

In response to various chronic insults, renal interstitial resident fibroblasts undergo morphological and functional alteration to transdifferentiate into myofibroblasts, which express α-SMA and produce abundance of extracellular matrix components^1,2^. Regardless of the origins of the myofibroblasts, uncontrolled fibroblasts activation and proliferation play a central role in the development of renal fibrosis^3,4^. However, the mechanisms underlying the fibroblasts' transdifferentiation into myofibroblasts are not completely understood.

Mitochondria are extremely dynamic organelles, constantly undergoing antagonistic processes of fission and fusion. Multiple mitochondrial membrane GTPases regulate mitochondrial dynamics. Mitofusin-1 (Mfn1), Mitofusin-2 (Mfn2), and optic atrophy 1 (OPA1) proteins mediate fusion, whereas dynamin-related protein 1 (Drp1) executes fission by recruiting to the mitochondrial outer membrane to drive scission^5–7^. Drp1 activity can be reversibly modified by two critical phosphorylation sites. Phosphorylation of Drp1 at serine 616 (p-Drp1S616) promotes Drp1 activity. Conversely, phosphorylation of serine 637 (p-Drp1S637) represses its activity and leads to mitochondrial elongation^5^. Beyond the primary function in remodeling mitochondrial structures, balanced mitochondrial dynamics play a vital role in maintaining mitochondrial function and cellular homeostasis, and abnormal mitochondrial dynamics are associated with the pathogenesis of several diseases, such as tumor, diabetes mellitus, and pulmonary fibrosis^5,7–10^.

Excessive fission has been reported in acute kidney injury and diabetic nephropathy^11–13^. Intensified Drp1 activation resulted in increased fragmentation and mitochondrial oxidative stress, promoting apoptosis in renal proximal tubular cells and podocytes^14,15^. However, whether and how mitochondrial dynamics in fibroblasts contributes to renal fibrosis has not been investigated. Recently, a study suggests metabolic shift from oxidative phosphorylation to glycolysis in myofibroblasts during the development of renal fibrosis^16^. In addition, mitochondrial dynamics are adaptive mechanisms for metabolic demands in a range of cells and tissues, and metabolic reprogramming has emerged as a key mechanism controlling cell division and proliferation, such as cancer cells, T effect cells, et al.^7,17–20^, that indicates the potential participation of mitochondrial dynamics in fibroblast to myofibroblast transition in injured condition.

Here, we demonstrated the increased mitochondrial fission in fibroblasts of fibrotic kidney. p-Drp1S616-mediated mitochondrial fission in fibroblasts caused mtROS elevation, glycolytic shift, and acetylation of H3K27, thereby leading to fibroblast activation and renal fibrogenesis via epigenetic regulation of fibrosis-related genes transcription. Thus, modulation of fibroblast mitochondrial dynamics may represent a novel therapeutic strategy for kidney fibrosis.

## Materials and methods

### Human kidney tissues

Human kidney tissues were obtained from the department of nephrology, the First Affiliated Hospital of Sun Yat-Sen University. The severity of interstitial fibrosis was evaluated by the Masson trichrome staining and estimated according to previously reported^21^. The characteristics of these patients at initial biopsy were described in Supplementary Table 1. The study was approved by the First Affiliated Hospital of Sun Yat-Sen University Institutional Review Boards (Guangzhou, China). All patients gave their written informed consent.

### Animals

Male C57BL/6J mice of 8 weeks were purchased from Beijing Vital River Laboratory Technology and housed in a specific-pathogen-free facility. Unilateral ureteral obstruction (UUO) surgery was conducted as described previously^22^. For inhibition of mitochondrial fission, 50 mg/kg mdivi-1 (Cay15559, Cayman, USA) was given by intraperitoneal injection 24 h before operation and then twice daily during the whole study period as previous reported^20^. To explore the therapeutic potential of mdivi-1 in established renal fibrosis, mdivi-1 was also administered to mice starting on day 3 after UUO until sacrifice at day 10. All animal experiments were performed with the approval of the Institutional Animal Care and Use Committee of the Sun Yat-Sen University.

### Renal histology and transmission electron microscopy examination

Paraffin-embedded 3-μm kidney sections were stained with Masson trichrome (BA4079A, Baso, China) and Sirius red (ab150681, Abcam, UK) as previously described^23^. Electron microscopic tissue samples handling and process were conducted by the electron microscopic core lab of Sun Yat-sen University. Lower magnification (×2000) was firstly used to identify interstitial fibroblasts or myofibroblasts, which featured by a spindle-shaped morphology, dense rough endoplasmic reticulum, bundled microfilaments, but a paucity of lysosomes^24^. Individual fibroblasts were then examined under higher magnification (×15000) to identify mitochondrial morphology and at least ten fields per tissue copper section were taken into analysis under 2000× magnification. For chronic kidney disease (CKD) patients, a total of 40 mitochondria in 23 fibroblasts from patients (*n* = 23) without tubulointerstitial fibrosis, 52 mitochondria in 20 fibroblasts and 69 mitochondria in 30 fibroblasts from patients with moderate (*n* = 10) and severe tubulointerstitial fibrosis (*n* = 10) were included in quantitative analysis. For UUO mice, 42 mitochondria in 25 fibroblasts and 146 mitochondria in 50 fibroblasts from sham-operated (*n* = 6) and UUO-operated kidneys (*n* = 6) were included in quantitative analysis, respectively. The mitochondrial length and aspect ratio (AR, ratio between the major and minor axis) were determined using NIH image J software (NIH, Bethesda, USA) from individual mitochondria, as previously reported^20^. Histopathology was evaluated by two pathologists in a double-blind manner.

### Cell culture and treatments

Normal rat kidney fibroblast cells (NRK-49F) were purchased from American Type Culture Collection. Cells were treated with 10 ng/ml TGF-β1 (240-B-002, R&D Systems, USA) for various time periods in the presence or absence of mdivi-1, siRNA, wild-type or mutant of Drp1 plasmid (FulenGen, China). Transfections were performed using Lipofectamine 3000 (L3000015, Invitrogen, USA) according to manufacturer’s protocols. Briefly, the siRNA (100 nm) or plasmid (2 μg) were mixed with 3 μl of Lipofectamine 3000 in 200 μl of Opti-mem (31985070, Gibco, USA) to form a liposome/nucleic acid complex; the complex was then added to cells with medium for transfection.

### Mitochondrial morphology analysis in vitro

NRK-49F cells were stained with 100 μM MitoTracker Red (M7512, Molecular probe, USA) in a dish at 37 °C for 20 min. The cell nuclei were stained with Hochest33342 and images were taken by a confocal microscope (Zeiss LSM510, Germany) at ten randomized fields per dish for further analysis. For quantitative evaluation of mitochondrial dynamic changes, AR (major axis/minor axis) and form factor (FF, (perimeter^2^)/(4*π* × surface area)) as previously reported^25^. Since the mitochondria within a cell were often either filamentous or fragmented, we classified the cells as mitochondrial fission based on the majority (>70%) of mitochondria fragmented, as defined as the mitochondrial length < 2 μm as previous reported^11^.

### Measurement of oxygen consumption rate and extracellular acidification rate

Oxygen consumption rate (OCR) and extracellular acidification rate (ECAR) were determined by using the Seahorse XFe96 Extracellular Flux Analyzer (Agilent, CA). Briefly, cells were seeded in XF96 Cell Culture Microplates and incubated at 37 °C for 24 h. The cultured medium was changed to XF assay medium supplemented with 1 mM pyruvate, 2 mM glutamine, and 10 mM glucose for OCR assay, or 1 mM glutamine for ECAR assay and placed in a 37 °C incubator without CO_2_ for an hour. OCR was measured by sequential injections of 1 µM oligomycin, 0.5 µM carbonyl cyanide 4-(trifluoromethoxy) phenylhydrazone (FCCP), and 1 µM rotenone plus antimycin A to perform a mitochondrial stress test using the XF Extracellular Flux Analyzer. ECAR was examined in basal conditions and during the sequential injection of 10 mM glucose, 1 µM oligomycin, and 50 mM 2-DG. After each assay, cells were lysed, and the protein concentration was measured to normalize the OCR and ECAR.

### Assessment of mitochondrial superoxide ROS production

Superoxide indicator, MitoSOX Red (M36008, Invitrogen, USA) was used to examine the mitochondrial reactive oxygen species (mtROS) production. Briefly, cells were incubated with 5 μM MitoSOX reagent for 10 min at 37 °C in the dark. Images were taken under a confocal microscopy (Zeiss LSM510; Carl Zeiss, Germany). The data were expressed as the percent of fluorescence generated in the controls.

### Detection of apoptosis and proliferation

Annexin V-FITC/PI staining was performed to quantify apoptotic cells as previously described^26^. Briefly, NRK-49F cells were collected and stained with annexin V-FITC and PI working solution (11858777001, Roche, Switzerland) according to the manufacturer’s protocol. The cells were analyzed using flow cytometer with 488 nm excitation and 530/570 nm emission. Cell proliferation was examined by 5-ethynyl-2′-deoxyuridine (Edu) incorporation using a Click-iT Edu Imaging Kit (C10337, Thermo Fisher Scientific, USA). Images were taken at ten randomized fields under the confocal microscope. The quantification was assessed by the proportion of cells with Edu-positive staining.

### Immunoblot analysis, immunofluorescence, and immunochemical staining

Kidney cortex or cells were lysed in RIPA buffer. Protein samples were separated by SDS-PAGE and transferred to polyvinylidene difluoride membranes. The membrane was incubated with the primary antibody at 4 °C overnight followed by horseradish-peroxidase (HRP)-conjugated secondary antibody. Primary antibodies used in this study were as follows: α-SMA (ab32575), Collagen I (ab34710), and PCNA (ab92552) purchased from Abcam (UK); Drp1 (8570), Drp1S616p (3455), and H3K27ac (8173) were purchased from Cell Signaling Technology company (USA).

Immunofluorescence staining was performed as previously described^22^. Briefly, paraffin-embedded kidney sections were treated with antigen retrieval solution, blocked, and incubated with the indicated primary antibodies followed by incubating with secondary antibodies-conjugated with Alexa Fluor 488 or 546, respectively. The percentage of positive staining was analyzed and quantified with ImageJ software as previous reported^23^.

For the immunochemical staining, after the paraffin-embedded kidney sections were dewaxed and antigen retrieved, the sections were blocked with 0.3% H_2_0_2_ in methanol and 5% BSA, respectively. Then, the sections were incubated with the indicating primary antibody at 4 °C overnight. After incubation with the HRP-conjugated secondary antibody, the DAB staining was conducted according to the manufacturer’s instructions (K50007, Dako, USA).

### Chromatin immunoprecipitation assays and real-time PCR analyses

Chromatin immunoprecipitation (ChIP) was performed using Chromatin Immunoprecipitation Assay Kit (17–295, Millipore, USA) according to the manufacturer’s protocol. Briefly, cells were cross-linked with 1% formaldehyde and lysed. Chromatin samples were isolated, sonicated to 200–500 bp and immunoprecipitated with antibodies against H3K27ac or a negative control normal rabbit IgG. Immunoprecipitated DNA fragments were purified using Qiagen DNA purification kit and quantified by real time PCR with primer pairs specific for the promoters of *α-SMA* and *PCNA* gene. The indicated primers were listed as follows: α-SMA: forward, 5′-GACTTCATTGATACTACACACA-3′, reverse, 5′-GTGGGTGGTGTCTGGGGAGGCTGA-3′; PCNA: forward, 5′-CAGAGCGAAGCACCCAGGTAAGT-3′, reverse, 5′-GGTACCCCGA CTCACGATGC AG-3′.

### Statistical analysis

Data are presented as mean ± SEM. Student’s *t*-test was used for statistical analysis between two groups; one-way analysis of variance (ANOVA) followed by Tukey’s post-test for multiple comparisons was used to determine significant differences for groups of three or more. *p* Values < 0.05 were considered statistically significant.

## Results

### Mitochondrial fission is enhanced in interstitial fibroblasts from fibrotic kidneys

**Fig. 1:**
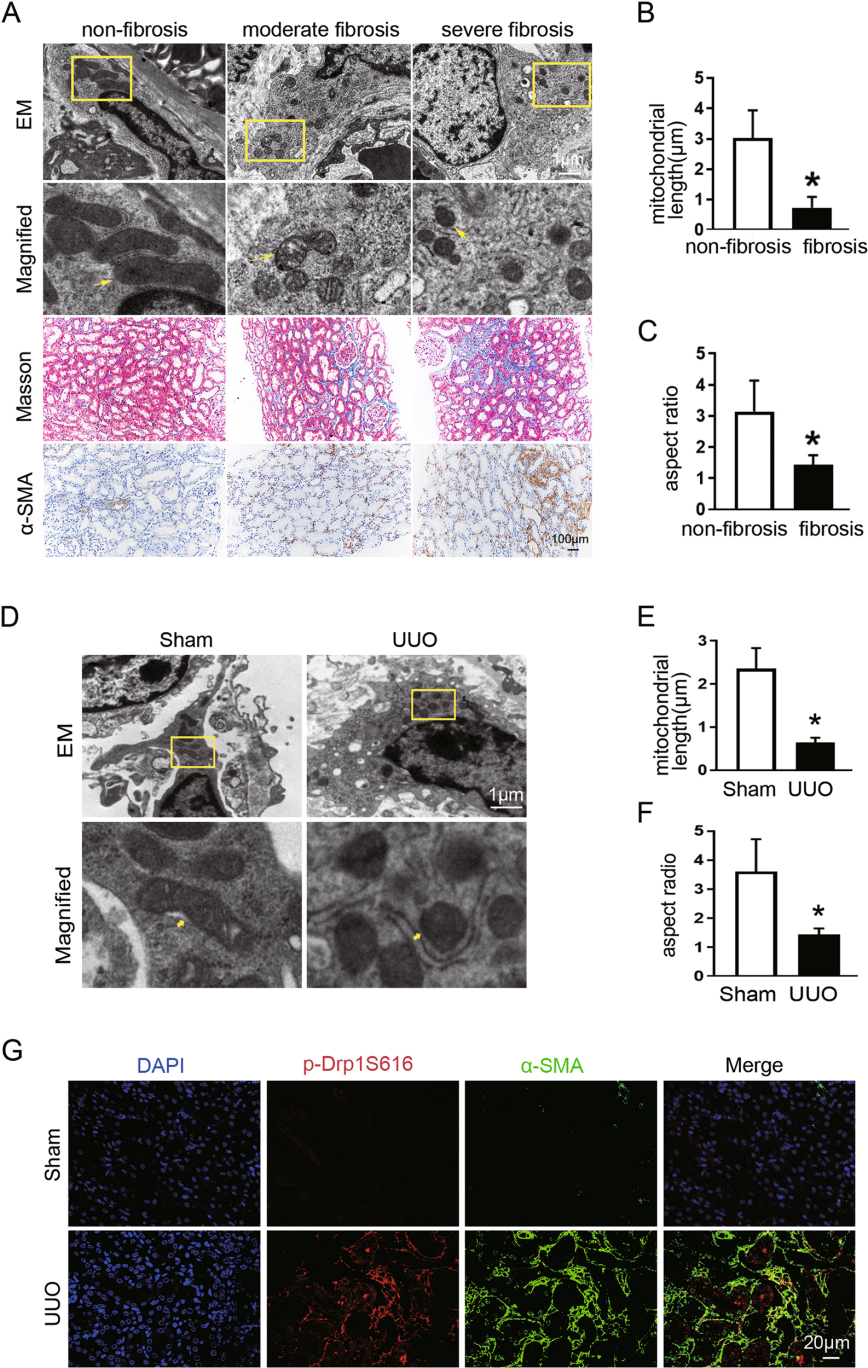
Mitochondrial fission is increased in interstitial fibroblasts in fibrotic kidneys from CKD patients and UUO mice. **a** Representative electron micrographs of mitochondrial morphology in fibroblasts, Masson staining, and the α-SMA immunochemical staining of renal sections from patients with different degree of renal fibrosis. Yellow arrows indicate mitochondria. **b** Quantitative analysis of mitochondrial length in fibroblasts among groups as indicated. **c** Quantification of mitochondrial aspect ratio in fibroblasts in each group. Data in **b** and **c** are means ± SEM (*n* = 23 in non-fibrosis group and *n* = 20 in fibrosis group); *p < 0.05 vs non-fibrosis. **d** Representative electron microscopy images of fibroblast mitochondria in sham and UUO kidneys. Yellow arrows indicate mitochondria. **e** Quantitative data showing mitochondrial length. **f** Quantitative analysis of mitochondrial aspect ratio. **g** Representative immunofluorescence staining of Drp1S616p (red) and α-SMA (green) in kidneys from mice subjected to sham or UUO operation. Data in **e** and **f** are means ± SEM (*n* = 6 per group); *p < 0.05 vs sham.

We first investigated the morphology of mitochondria in interstitial fibroblasts in patients with different stages of chronic kidney disease. Transmission electron microscopy (TEM) revealed that compared with nonfibrotic kidneys, mitochondria were rounder and smaller in the fibroblast of fibrotic kidneys, indicating impaired mitochondrial dynamics. The mitochondrial morphological changes and increased expression of α-SMA corresponded to fibrosis severity detected by Masson’s trichrome staining and immunochemical staining (Fig. 1a). Quantitative analysis of mitochondrial morphology in fibroblasts demonstrated that average mitochondrial length decreased from 2.93 ± 0.90 μm to 0.72 ± 0.35 μm (Fig. 1b) and AR from 3.14 ± 0.99 to 1.42 ± 0.31 (Fig. 1c) in nonfibrotic and fibrotic kidneys, respectively. These results indicate that impaired mitochondrial dynamics in fibroblasts may be involved in the pathogenesis of renal fibrosis.

We then analyzed fibroblast mitochondrial dynamics in the UUO mice model. Similar to human kidney tissues, interstitial fibroblasts exhibited long filamentous mitochondria in sham-operated kidney. In contrast, mitochondria became split, fragmented, or punctate, together with abundant cytoplasm and increased rough endoplasmic reticulum in the fibroblast of UUO kidney (Fig. 1d). Quantitative determination revealed that the length of mitochondria was significantly reduced from 2.35 ± 0.47 to 0.63 ± 0.11 (Fig. 1e) and AR from 3.60 ± 1.15 to 1.41 ± 0.19 (Fig. 1f) in fibroblasts of UUO kidneys, as compared with those in sham-operated kidneys. Given the importance of Drp1 in mitochondrial fission^27,28^, we further examined its protein levels and p-Drp1S616 in kidneys. Immunofluorescence staining results showed that the expression of α-SMA was barely expressed and p-Drp1S616 was not detectable in sham-operated kidneys, whereas increased expression of p-Drp1S616 and α-SMA positive myofibroblasts accumulation was observed in UUO-induced fibrotic kidneys (Fig. 1g). Collectively, these data indicate that Drp1 appears to involve in regulating fibroblast mitochondrial fission during renal fibrosis.

### Pharmacologic inhibition of Drp1 reduces fibroblast accumulation and renal fibrosis

**Fig. 2:**
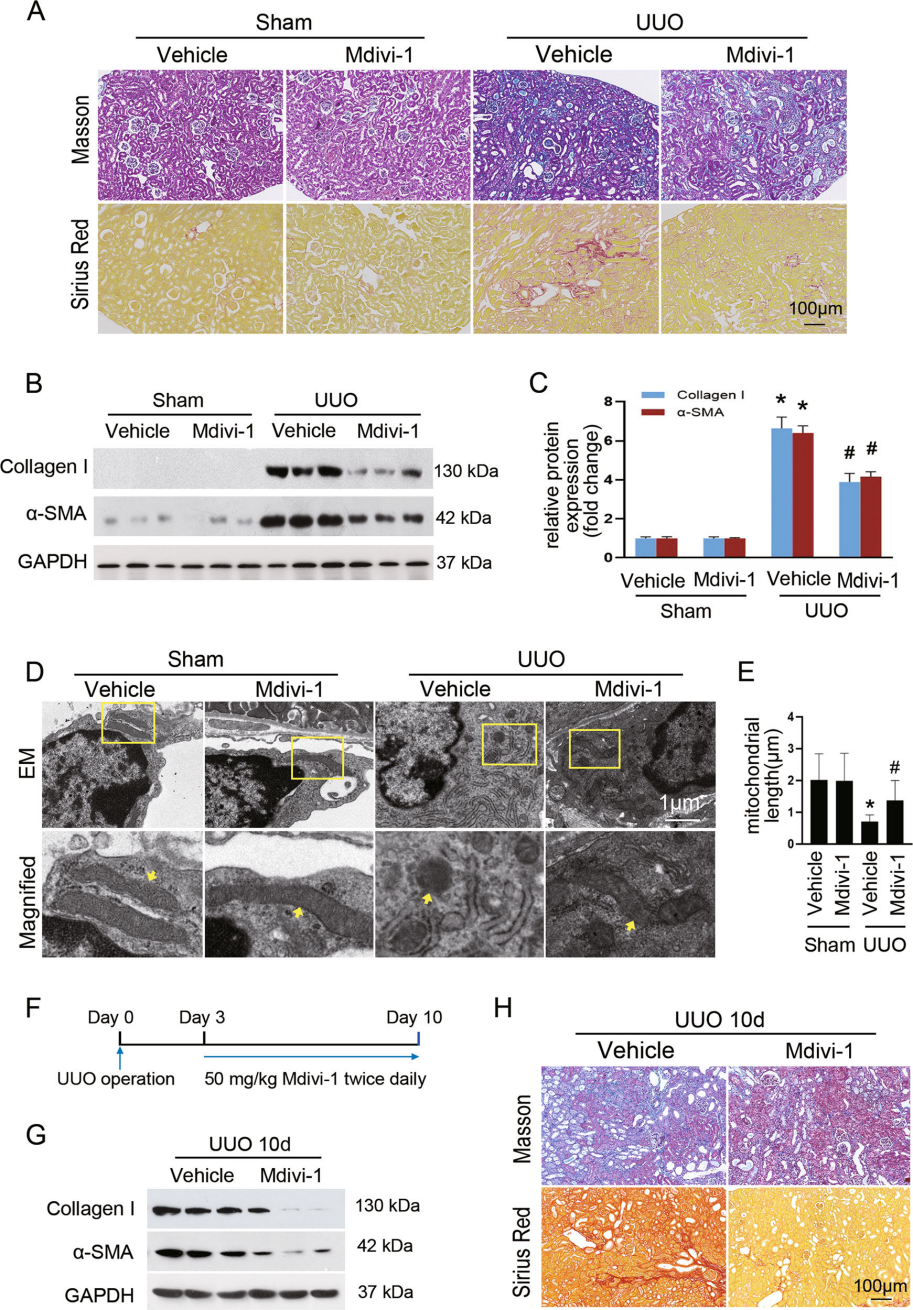
Suppression of mitochondrial fission by Mdivi-1 alleviates renal fibrosis. **a** Masson’s trichrome (upper panels) or Sirius red staining (lower panels) of renal sections among indicated groups. **b** Immunoblot analyses showing the expression of collagen I and α-SMA in the fibrotic kidneys compared with sham control. **c** Densitometry analyses of collagen I and α-SMA in immunoblots. GAPDH was used as a loading control. **d** Representative electron micrographs of mitochondria from renal interstitial fibroblasts among groups as indicated. **e** Quantitative analysis of mitochondrial length. **f** Experimental design of evaluating the therapeutic effect of mdivi-1 on established fibrosis. **g** Immunoblot analyses showing the expression of collagen I and α-SMA in the mdivi-1-treated fibrotic kidneys compared with vehicle control. **h** Masson’s trichrome (upper panels) or Sirius red staining (lower panels) of kidney sections of the indicated groups. Data in **c** and **e** are means ± SEM (*n* = 6 per group); *p < 0.05 vs sham groups; #*p* < 0.05 vs vehicle-treated obstructed groups.

Based on above findings, we explored whether imbalance of mitochondrial fusion and fission in fibroblasts is an early event during the development of renal fibrosis or occurred as consequence of the pathological state triggered by cellular injury. To this end, mice were treated with mdivi-1, an inhibitor of mitochondrial fission protein Drp1, or vehicle during UUO. Masson’s trichrome and picrosirius red staining showed that mdivi-1-treated mice had a marked reduction in collagen deposition and fibrosis 7 days after UUO compared to those treated with vehicle (Fig. 2a). Consistent with histological observation, immunoblot analysis revealed mdivi-1 significantly attenuated UUO-induced expression of α-SMA and collagen I (Fig. 2b, c). By electron microscopy (EM), we confirmed that UUO-induced fibroblast mitochondrial fragmentation was partially blocked by mdivi-1 treatment (Fig. 2d, e). Moreover, mdivi-1 treatment was able to inhibit the established renal fibrosis (Fig. 2f, g). These results suggest that suppression of Drp1-mediated mitochondrial fission attenuates UUO-induced renal interstitial fibrosis.

### Inhibition of Drp1-mediated mitochondrial fission attenuates TGF-β-induced fibroblast activation in vitro

To provide direct evidence that links mitochondrial fission to fibroblast activation, we stimulated NRK-49F cells with TGF-β1 as previously reported^23^ and performed immunofluorescence staining with Mitotracker Red, a fluorescence probe for mitochondria labeling. We found that mitochondria became short and punctate (Supplementary Fig. 1A), around 80% cells displayed fragmented mitochondria after 48 h of TGF-β1 treatment (Supplementary Fig. 1B–D). Further, the amount of p-Drp1S616 increased over time that was pronounced at 6 h after TGF-β1 stimulation, with no significant alteration in the levels of total Drp1 and reduced content of p-Drp1S637. Meanwhile, there was no significant change of Mfn2 and OPA1 expression in TGF-β1-treated cells (data not shown). In agreement with our previous report^23^, TGF-β1 induced upregulation of α-SMA expression in a time-dependent manner (Supplementary Fig. 1E, F).

**Fig. 3:**
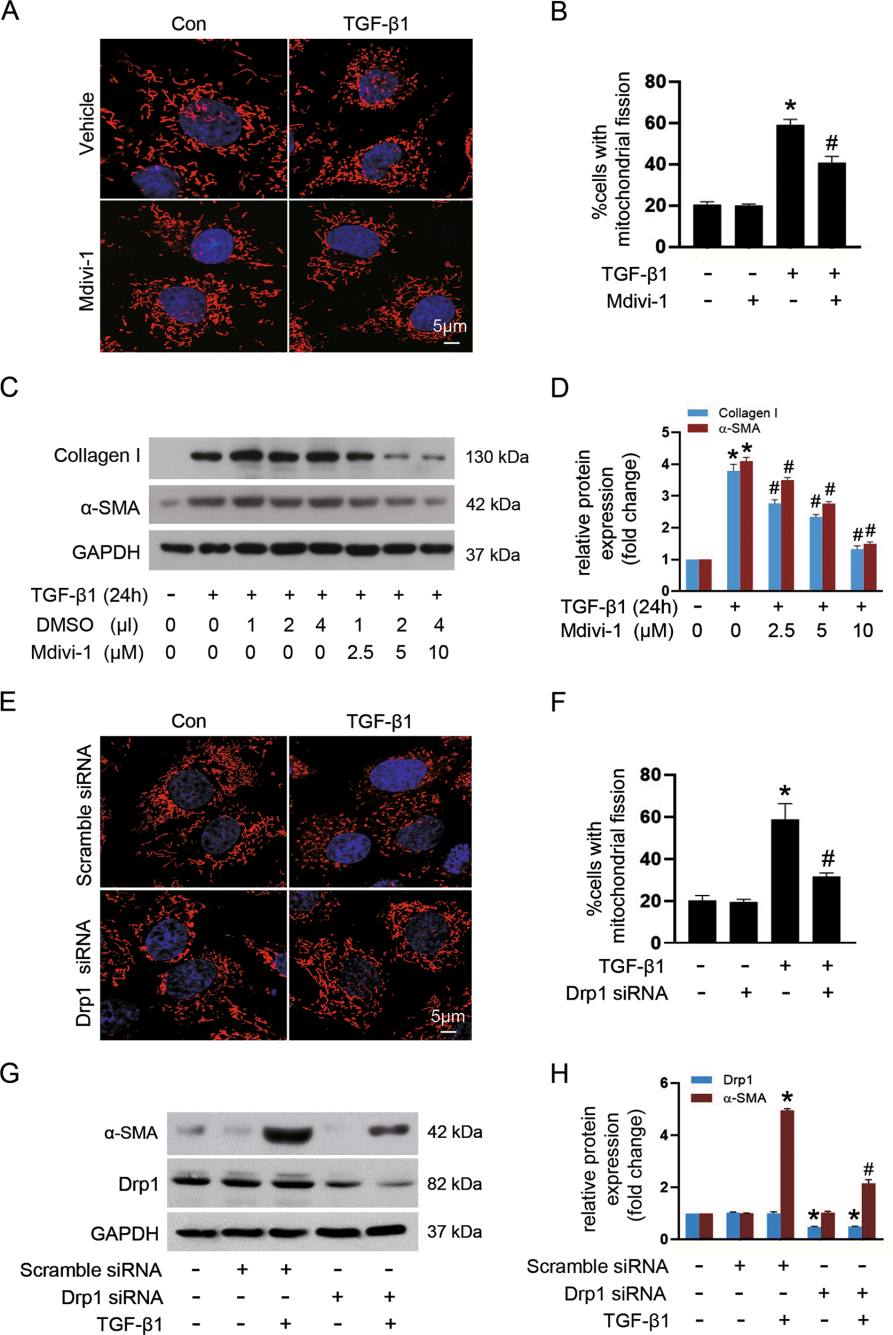
Targeting Drp1-mediated mitochondrial fission blocks TGF-β-induced fibroblast activation in vitro. NRK-49F cells were pretreated with Mdivi-1 or transiently transfected with either Drp1 siRNA or scramble siRNA, followed by stimulation with 10 ng/ml TGF-β1 for 24 h. **a** Representative images of mitochondria stained with MitoTracker Red in cells among indicated groups. **b** Quantification of the percentage of cells displaying fragmented mitochondria. **c** Immunoblot analyses of collagen I and α-SMA in cells treated with or without mdivi-1 and exposure to TGF-β1. **d** Densitometric analysis of immunoblots in **c**. **e** Representative images of fibroblasts subjected to MitoTracker (red) and DAPI (blue) staining among indicated groups. **f** Quantitative analyses for the percentage of cells displaying fragmented mitochondria. **g** Immunoblot analyses of Drp1 and α-SMA protein expression in fibroblasts among different groups. **h** Relative expression levels of Drp1 and α-SMA normalized to GAPDH by densitometry. Data in **b**, **d**, **f**, and **h** are expressed as means ± SEM (*n* = 3); *p < 0.05 vs TGF-β1-untreated cells; #*p* < 0.05 vs TGF-β1-treated cells alone or with scramble siRNA.

Given the protective role of mdivi-1 in UUO-associated renal fibrosis, we next sought to explore the possibility that Drp1 might directly regulate fibroblast activation in vitro. We found that mdivi-1 markedly decreased TGF-β1-triggered fibroblast mitochondrial fission detected by Mitotracker Red (Fig. 3a) from 59.2% to 40.8% cells with fragmented mitochondria (Fig. 3b). Moreover, Mdivi-1 reduced TGF-β1-elicited expression of α-SMA and collagen I, and these effects were dose-dependent (Fig. 3c, d). To verify the potential role of Drp1-mediated mitochondrial fission in fibroblast activation, NRK-49F cells were transfected with Drp1 siRNA or scramble siRNA. Compared with scramble siRNA control, Drp1 siRNA led to a dramatic decrease in mitochondrial fission from 58.8% to 31.8% cells with fragmented mitochondria upon TGF-β1 treatment (Fig. 3e, f). Accordingly, the expression of α-SMA was markedly downregulated in cells transfected with Drp1 siRNA (Fig. 3g, h). These results provided direct evidence that Drp1-mediated mitochondrial fission regulates fibroblast activation.

### Drp1 S616 phosphorylation is necessary for TGF-β-induced fibroblast activation and proliferation

**Fig. 4:**
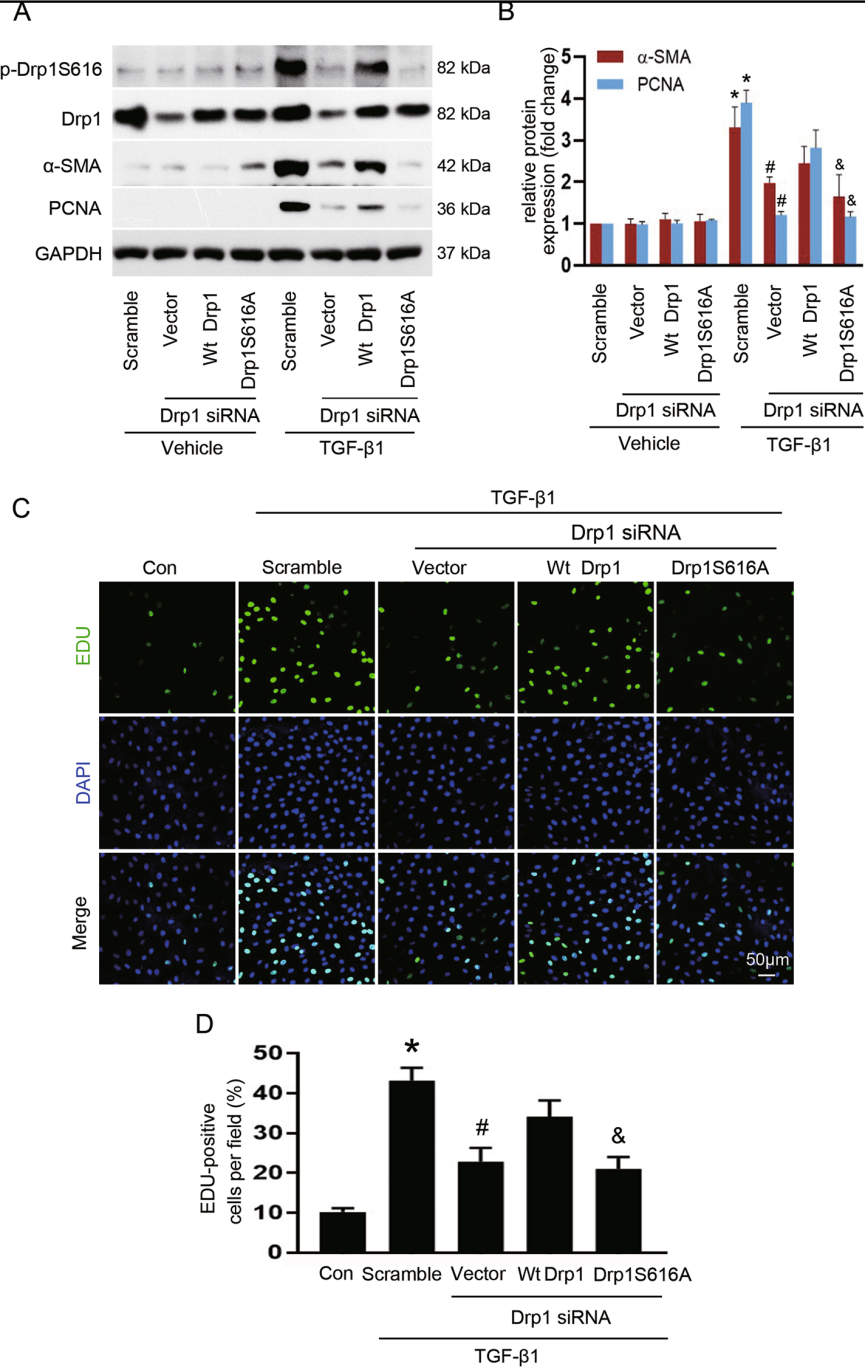
Drp1S616 phosphorylation controls TGF-β-induced fibroblast activation and proliferation. NRK-49F cells were treated as described in the Materials and methods section. **a**, **b** Representative immunoblot (**a**) and quantitative analyses (**b**) of α-SMA and PCNA in cells among different groups. Data are means ± SEM (*n* = 3); *p < 0.05 vs vehicle; #*p* < 0.05 vs TGF-β1-treated cells transfected with scramble siRNA; &*p* < 0.05 vs TGF-β1-treated cells co-transfected with Wt Drp1 and Drp1 siRNA. **c**, **d** Representative images of EDU incorporation (green) reflecting cell proliferation (**c**) and quantification of EDU-positive cells (**d**). Data are means ± SEM (*n* = 3); *p < 0.05 vs control; #*p* < 0.05 vs TGF-β1-treated cells transfected with scramble siRNA; &*p* < 0.05 vs TGF-β1-treated cells co-transfected with Wt Drp1 and Drp1 siRNA.

Phosphorylation of Drp1 at serine 616 promotes mitochondrial fission that is closely related to the mitosis, facilitating the mitotic cell cycle^29^. To determine whether p-Drp1S616 is required for TGF-β1-induced fibroblast activation and proliferation, NRK-49F cells with endogenous Drp1 knockdown were further transfected with empty vector, wild-type Drp1 (Wt Drp1), or phosphorylation-deficient mutant of Drp1 (Drp1S616A), respectively. As shown in Fig. 4a, b, in control cells without Drp1 siRNA transfection (lanes 1 and 5), TGF-β1 stimulation led to increased expression of p-Drp1S616, α-SMA, and PCNA compared to vehicle control. In Drp1 depletion cells, with or without TGF-β1 exposure, re-expression of either Wt Drp1 or Drp1S616A potently rescued Drp1 protein levels when compared with empty vector control (lanes 3 and 4 versus lane 2, lanes 7 and 8 versus lane 6). Transfected with Drp1 siRNA alone or co-transfected with Drp1 siRNA and mutant Drp1S616A virtually repressed TGF-β1-induced fibroblast activation and proliferation, as indicated by blockage of the expression of α-SMA and PCNA, respectively (lanes 6 and 8 versus lane 5). However, Drp1 deletion cells re-expression with Wt Drp1 failed to inhibit the above effects induced by TGF-β1, in accordance with increased level of p-Drp1S616 (lane 7 versus lane 5). Accordingly, Drp1 knockdown only or Drp1 knockdown with re-expression of Drp1S616A, but not Wt Drp1, retarded TGF-β1-induced fibroblast proliferation compared to cells transfected with scramble siRNA, as evidenced by a significantly reduction in the EDU-positive cells (Fig. 4c, d). Together, our data suggest that mitochondrial fission facilitates fibroblast activation and proliferation, which is dependent on Drp1 S616 phosphorylation.

### Drp1 knockdown alleviates TGF-β-induced mitochondrial dysfunction, mtROS elevation, and apoptosis-resistance in fibroblasts

**Fig. 5:**
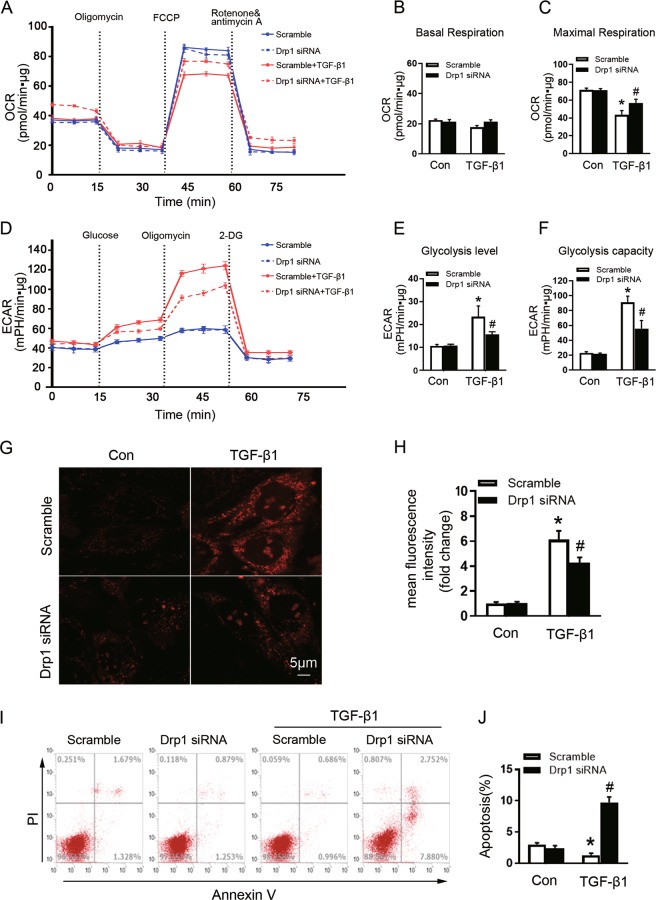
Knockdown of Drp1 reverses TGF-β-induced fibroblast mitochondrial dysfunction and apoptosis resistance. **a**–**c** Measurements of the OCR and **d**–**f** the ECAR metabolic profile in NRK-49F cells expressing either Drp1 siRNA or scramble siRNA and exposed to TGF-β1, as described in the Materials and methods section. Mitochondrial basal respiration (**b**), mitochondrial respiration capacity (**c**), glycolytic level (**e**), and glycolysis capacity (**f**) of cells among different groups as indicated. **g** Representative graphs of MitoSOX red stained in cells among different groups as indicated. **h** Analysis of MitoSOX Red fluorescence intensity among different groups as indicated. **i** The apoptosis was examined by flow cytometry analysis among groups as indicated. **j** The quantification analysis of the percentage of apoptotic cells. Data in **b**, **c**, **e**, **f**, **h**, and **j** are means ± SEM (*n* = 3); *p < 0.05 vs control cells; #*p* < 0.05 vs TGF-β1-treated cells with scramble siRNA.

Because the loop between mitochondrial dynamics and bioenergetics impacts cell fate^19,30,31^, we next examined the role of Drp1 in mitochondrial bioenergetic function in NRK-49F cells. Drp1 knockdown did not affect the basal mitochondrial OCR, regardless of TGF-β1 exposure for 24 h (Fig. 5a, b). The maximal respiration declined in scramble siRNA-transfected cells upon TGF-β1 stimulation, and this response was partially reversed in Drp1 siRNA-treated cells (Fig. 5a, c). The ECAR analysis indicated that Drp1 knockdown significantly conquered TGF-β1-triggered increase in glycolysis level (Fig. 5d, e) and glycolytic capacity (Fig. 5d, f). Further, Drp1 depletion dramatically reduced TGF-β1-induced mtROS generation without affecting its production under physiological condition (Fig. 5g, h). These data suggest that Drp1 positively regulates TGF-β1-induced glycolysis and mtROS production in renal fibroblasts.

Metabolic reprogramming toward glycolysis has been implicated in promoting apoptosis resistance in cancer cells^32–34^; we next assessed whether Drp1 is involved in TGF-β1-induced apoptosis. NRK-49F cells transfected with scramble siRNA exhibited reduced apoptosis, indicating fibroblast resistance to TGF-β1-induced apoptosis. In contrast, Drp1 knockdown significantly conquered TGF-β1-induced fibroblast apoptosis tolerance, with enhancing apoptosis from 1.78% to 9.67% (Fig. 5i, j). Thus, our data suggest that Drp1 ablation potentiates mitigation of TGF-β1-induced fibroblast proliferation partially through increasing apoptosis.

### Drp1 facilitates fibroblast activation and proliferation by acetylating H3K27 at the promoters of α-SMA and PCNA

**Fig. 6:**
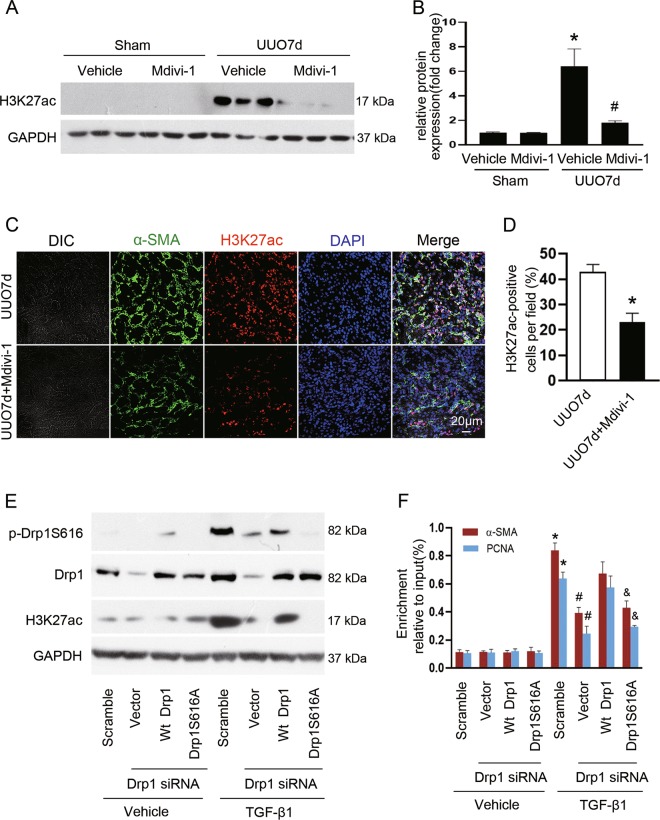
Drp1 facilitates H3K27ac binding at the promoters of α-SMA and PCNA induced by TGF-β1. **a** Kidney tissue lysates were subjected to immunoblot analysis using antibodies against H3K27ac and GAPDH. **b** The expression level of H3K27ac was quantified by densitometry and normalized with GAPDH. Data are means ± SEM (*n* = 6 per group); *p < 0.05 vs sham groups; #*p* < 0.05 vs vehicle-treated obstructed groups. **c** Representative immunofluorescent staining of α-SMA (green) and H3K27ac (red) in kidneys from mice subjected to UUO operation and treated with or without Mdivi-1. Nuclei (blue) were stained with DAPI. **d** Quantification of cells with H3K27ac positive. Data are means ± SEM (*n* = 6 per group); *p < 0.05 vs mice treated with vehicle. **e** Immunoblot analyses of the indicated proteins from NRK-49F cells after various treatment. **f** Chromatin immunoprecipitation assay was performed with H3K27ac antibody using nuclear extracts harvested from NRK-49F cells after various treatment. The immunoprecipitated DNA fragments were amplified by PCR using primers specific for α-SMA and PCNA promoter. Data are means ± SEM (*n* = 3); *p < 0.05 vs vehicle; #*p* < 0.05 vs TGF-β1-treated cells transfected with scramble siRNA; &*p* < 0.05 vs TGF-β1-treated cells co-transfected with Wt Drp1 and Drp1 siRNA.

Metabolism reprogramming regulated by mitochondrial remodeling may affect histone acetylation, and H3K27ac is a well-defined marker of active enhancers^35,36^. We thus explored the impact of mitochondrial fission on H3K27ac. Our results showed that H3K27ac was significantly increased in the obstructed kidneys of vehicle-treated mice, which was dramatically suppressed by mdivi-1 (Fig. 6a, b). Double immunostaining further revealed that renal interstitial cells expressing α-SMA also expressed H3K27ac in vehicle-treated UUO mice, while mdivi-1 markedly attenuated this response (Fig. 6c, d). To address the role of phosphorylated Drp1 for regulating H3K27 acetylation and gene transcription, we performed rescue experiment by co-transfecting with Drp1 siRNA and empty vector, Wt Drp1, or Drp1S616A. We found that upon TGF-β1 treatment, H3K27ac was increased, as compared with control (Fig. 6e, lane 1 versus lane 5), overexpression of Wt Drp1, but not Drp1S616A, reversed H3K27 acetylation (Fig. 6e, lanes 7 and 8 versus lane 6) paralleled by increased binding of H3K27ac at the promoters of *α-SMA* and *PCNA* in Drp1 knockdown cells treated with TGF-β1 (Fig. 6f, bars 7 and 8 versus bar 6) as compared to cells transfected with empty vector, suggesting that p-Drp1S616-mediated mitochondrial fission may contribute to fibroblast activation and proliferation through the epigenetic regulation of gene transcription.

## Discussion

The present study demonstrated increased mitochondrial fission activity in interstitial fibroblast in clinical kidney fibrosis and experimental CKD model. Pharmacological inhibition of mitochondrial fission attenuated renal fibroblast activation in vitro and reduced renal fibrosis in vivo. Targeting Drp1 in cultured renal fibroblasts alleviated TGF-β1-induced mitochondrial dysfunction and apoptosis resistance. One of the principal mechanisms is that Drp1S616 phosphorylation is required for H3K27 acetylation and its binding at the promoter of *α-SMA* and *PCNA* to promote fibroblasts activation and proliferation. Our findings, for the first time, underscore a critical role of targeting Drp1-mediated mitochondria fission of fibroblasts in protecting against kidney fibrosis.

Elevated mitochondrial fission has been implicated in the progression of renal disease^11,14,15^. Suppression of mitochondrial fission by mdivi-1 has been proved to exert a cytoprotective effect in renal epithelial cells (TECs) in animal models of acute kidney injury^11^. In addition, Perry et al., by using TECs-specific Drp1 knockout mice, revealed a critical role of blockage of mitochondrial fission in preserving mitochondrial function and preventing fibrosis progression after acute kidney injury^14^. Furthermore, Danesh et al. have demonstrated that high-glucose-induced mitochondrial fission promotes pathogenesis of diabetic nephropathy by using podocyte-specific Drp1 knockout or Drp1S600A knockin mice^12,13^. Besides tubular cells or podocytes, it is noteworthy that resident fibroblasts could transdifferentiate into myofibroblasts and are major drivers of fibrosis, especially in the advanced stage of CKD. However, the alteration and roles of fibroblast mitochondrial dynamics have not been studied in kidney disease. In the present study, we showed that mitochondrial fission of fibroblasts was increased in renal biopsy samples of CKD patients and in tubulointerstitial fibrosis induced by UUO. Moreover, mdivi-1 mitigated interstitial myofibroblast accumulation and fibrosis in UUO kidney, suggesting that the anti-fibrosis effect of mdivi-1 may partly attribute to the suppression of fibroblast mitochondrial fission, and thereby inhibiting their activation and proliferation. Nevertheless, further studies by using the refined and specific genetic approach are needed to confirm our in vivo findings in the future.

The connection between mitochondrial fission and fibroblast activation is further supported by in vitro evidence. Drp1 deletion by Drp1 silence reduced TGF-β1-induced mitochondrial fission, as well as activation and proliferation in cultured fibroblast. Our results are consistent with previous studies showing that ablation of Drp1 dampens tumor growth and metastatic progression^7,17,18,37^, suggesting the detrimental influence of Drp1-mediated fibroblast mitochondrial fission in renal fibrosis. Further, by modulating Drp1 activity using phosphorylation-deficient mutant Drp1S616A, siRNA, or Wt-Drp1, we verified that fibroblast activation and proliferation induced by TGF-β1 was dependent on Drp1S616 phosphorylation. Notably, Drp1 siRNA sensitized, rather than halted, fibroblast apoptosis in response to TGF-β1, suggesting an anti-apoptosis function of Drp1 in renal fibroblasts. This result was contradictory to previous findings that Drp1 possessed pro-apoptosis property in TECs and podocytes^14,15^. In general, both TECs and podocytes mainly rely on mitochondrial oxidative phosphorylation to supply energy demand^12,38^. However, a study reported that oxidative phosphorylation to glycolytic gene shift occurred in renal stromal cells during fibrotic process, and inhibition of glycolytic shift suppressed the fibroblasts activation and proliferation^16^. Our results showed TGF**-**β1 stimulation increased mitochondrial fission along with glycolytic shift in fibroblasts, which was rescued by Drp1 depletion, suggesting an intrinsic connection between asymmetric mitochondrial fission and glycolysis in myofibroblasts. We also observed decreased maximal respiration capacity upon TGF-β1 stimulation, which was in line with the mitochondrial fission-mediated metabolism reprogramming in cancer cells^18^. Hence, the different phenotype of Drp1 inhibition between TECs/podocytes and fibroblasts was probably due to the discrepancy in intrinsic cellular metabolism pattern. However, the underlying mechanisms require further investigation. Taken together, our data reveal that renal interstitial fibroblasts with elevated mitochondrial fission are positively associated with myofibroblast differentiation and fibrosis, which may be attributed to the role of Drp1 in promoting fibroblast glycolytic shift, proliferation, and apoptosis resistance.

Increasing evidence demonstrates that metabolic reprogramming regulates myofibroblast differentiation in organ fibrosis^16,39^. The cellular metabolism is tightly linked to chromatin modification by adjusting local concentrations of key metabolites. It has been reported that glycolysis-mediated production of acetyl-CoA promotes histone acetylation and contributes to pluripotency maintenance in embryonic stem cells^40^. Plenty of studies have demonstrated the protective effect of histone acetylation inhibition in TECs during renal fibrosis^41,42^. However, it is unclear whether histone acetylation regulates fibroblasts activation. In the present study, elevated acetylation of H3K27 in myofibroblasts was observed in UUO kidney, which was predominantly expressed in α-SMA positive interstitial myofibroblasts. Inhibiting Drp1-mediated mitochondrial fission attenuated TGF-β1-induced acetylation of H3K27 and reduced its recruitment in promoters of *α-SMA* and *PCNA*, therefore leading to suppress fibroblast activation and proliferation. However, the molecular basis of Drp1 and/or mitochondrial dynamics in regulating histone modification remains to be investigated.

Collectively, our study has demonstrated a crucial role of increased mitochondrial fission in fibroblasts differentiating into myofibroblasts and renal fibrogenesis. Ablation of Drp1-mediated mitochondrial fission may represent a novel therapeutic strategy in preventing renal fibrosis.

## Supplementary information

Supplementary table 1

Supplementary figure 1

Supplementary figure legend

Author contribution form

Reporting checklist
