# Supplementary material for: Drp1-mediated mitochondrial fission promotes renal fibroblast activation and fibrogenesis
Source: Cell Death Dis. 2020 Jan 16;11(1):29. doi: 10.1038/s41419-019-2218-5 (PMC6965618; doi:10.1038/s41419-019-2218-5)
Supplement: Supplementary file 1 — Supplementary table 1 [file 41419_2019_2218_MOESM1_ESM.docx]

Supplementary table 1. Characteristics of the study patients among groups

| Variable | Non-fibrosis | Moderate fibrosis | Severe fibrosis | *P* value |
| --- | --- | --- | --- | --- |
| No. of patients | 23 | 10 | 10 | - |
| Age (year) | 22.30±3.81 | 35.20±5.22 | 39.10±8.08 | <0.001 |
| Male | 10 | 4 | 6 | 0.628 |
| Creatinine (μmol/l) | 69.87±10.23 | 180.10±38.90 | 305.90±69.87 | ＜0.001 |
| eGFR (ml/min/1.73m^2^) | 86.02±9.15 | 62.01±8.34 | 39.79±5.93 | ＜0.001 |
| Primary disease |  |  |  | - |
| MCD | 10(43.48%) | 0 | 0 | - |
| IgA nephropathy | 8(34.78%) | 5(50%) | 6(60%) | - |
| Membranous nephropathy | 0 | 3(30%) | 2(20%) | - |
| FSGS | 5(21.74%) | 2(20%) | 2(20%) | - |

Values shown as means ± SDs or median with frequencies (%).

Abbreviations: eGFR, estimated glomerular filtration rate; MCD, minimal change disease; FSGS, focal segmental glomerulosclerosis.
