# Supplementary figures and images for: Drp1-mediated mitochondrial fission promotes renal fibroblast activation and fibrogenesis
Source: Cell Death Dis. 2020 Jan 16;11(1):29. doi: 10.1038/s41419-019-2218-5 (PMC6965618; doi:10.1038/s41419-019-2218-5)

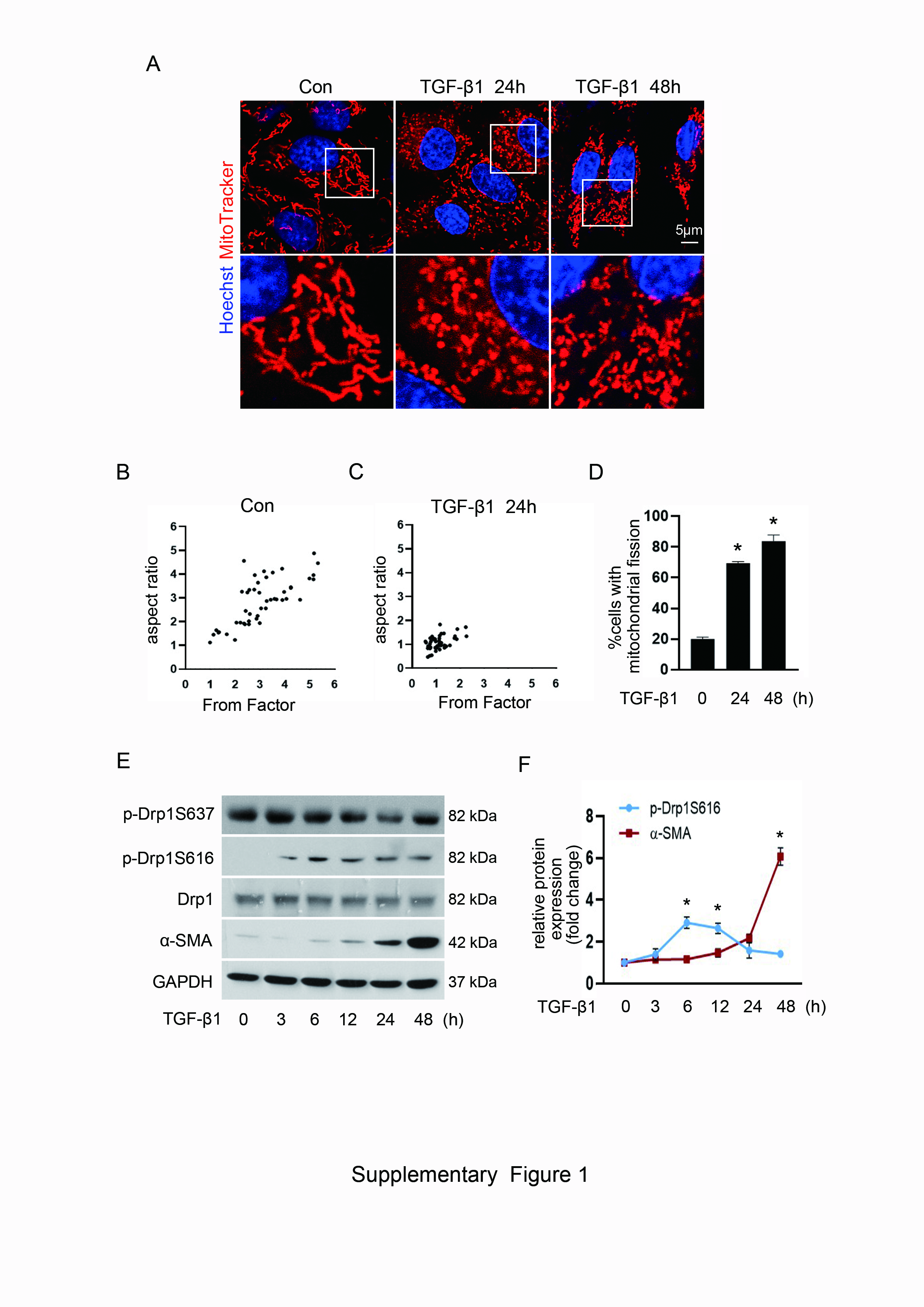

Supplement: Supplementary file 2 — Supplementary figure 1 [file 41419_2019_2218_MOESM2_ESM.tif]
