# Supplementary material for: Drp1-mediated mitochondrial fission promotes renal fibroblast activation and fibrogenesis
Source: Cell Death Dis. 2020 Jan 16;11(1):29. doi: 10.1038/s41419-019-2218-5 (PMC6965618; doi:10.1038/s41419-019-2218-5)
Supplement: Supplementary file 3 — Supplementary figure legend [file 41419_2019_2218_MOESM3_ESM.docx]

**Supplementary Fig. 1 mitochondrial fission is enhanced in TGF-β-induced fibroblast activation in vitro.** NRK-49F cells were treated with 10 ng/ml TGF-β1 for the indicated time. (A) Representative MitoTracker staining (red) of mitochondria in TGF-β1-treated cells. (B) Analysis of mitochondrial morphology by form factor and aspect ratio in control cells. (C) Analysis of mitochondrial morphology by form factor and aspect ratio in TGF-β1-treated cells. (D) Quantitative data showing the percentage of cells displaying fragmented mitochondria. (E) Immunoblot analyses of the indicated proteins expression from fibroblast treated with TGF-β1 for the indicated time. (F) Quantitative determination of the relative abundance of p-Drp1S616 and α-SMA among indicated groups. GAPDH was used to verify equivalent loading. Data in D and F are expressed as means ± SEM (n=3); * *p* <0.05 vs control.
