# Supplementary material for: Drp1-mediated mitochondrial fission promotes renal fibroblast activation and fibrogenesis
Source: Cell Death Dis. 2020 Jan 16;11(1):29. doi: 10.1038/s41419-019-2218-5 (PMC6965618; doi:10.1038/s41419-019-2218-5)
Supplement: Supplementary file 4 — Author contribution form [file 41419_2019_2218_MOESM4_ESM.pdf]

DECLARATION OF CONTRIBUTIONS TO ARTICLE

ADMC

Manuscript Number: CDDIS-19-3097RR

Journal Name: *Cell Death & Disease* (the 'Journal')

Proposed Title of the Contribution:

Drp1-mediated mitochondrial fission promotes renal fibroblast activation and fibrogenesis (the 'Contribution)

Author(s):

Yating Wang, Miaoqing Lu, Liping Xiong, Jinjin Fan, Yi Zhou, Huiyan Li, Xuan Peng, Zhong Zhong, Yihan Wang, Fengxian Huang, Wei Chen, Xueqing Yu, Haiping Mao (the 'Authors')

For all *CDDis* articles, each person named as an author in the published version must be able to show he or she has contributed substantially to the article.

Authorship credit should be based on 1) substantial contributions to conception and design, acquisition of data, or analysis and interpretation of data; 2) drafting the article or revising it critically for important intellectual content; and 3) final approval of the version to be published. Authors should meet conditions 1, 2 and 3.

Any person who cannot be shown to have made a substantial contribution to the article cannot be listed as an author in the final version. The name of any person who is deemed to have made a minor contribution can, however, appear in the Acknowledgments section of the article.

Please complete the table below to indicate the contributions of all named authors to the manuscript.

Author Full Name:	Specification of Contribution to the Manuscript:
Yating Wang	Yating Wang designed the study, did the most in vitro and in vivo study and wrote manuscript.
Miaoqing Lu	Miaoqing Lu performed partial in vivo experiment, assembled the figure and wrote manuscript.
Liping Xiong	Liping Xiong did the statistical analysis and wrote manuscript
Jinjin Fan	Jinjin Fan did the confocal image and microscope image collection
Yi Zhou	Professor Zhou did the in vivo experiments and gave material support.
Huiyan Li	Huiyan Li did partial in vitro study.
Xuan Peng	Xuan Peng did partial in vivo study.
Zhong Zhong	Zhong Zhong did partial in vivo study.
Yihan Wang	Professor Wang contributed to the electron microscope examination
Fengxian Huang	Professor Huang gave advise and revised the manuscript.
Wei Chen	Professor Chen commented and revised the article
Xueqing Yu	Professor Yu gave suggestion and revised the manuscript.
Haiping Mao	Professor Mao designed and supervised the study and revised the manuscript.

Please complete the table below to indicate the contributions of all named authors to the figures.

Figure 1:

Yating Wang, Miaoqing Lu and Liping Xiong did the mice UUO operation and collected the kidney tissue. Yating Wang collected the patients data, did the immunofluorescence and Masson staining and IHC staining, and helped transmission electron microscopy.

Figure 2:

Yating Wang did the transmission electron microscopy analysis and did the western blot experiments. Zhong Zhong and Xuan Peng did the Sirius and Masson staining. Professor Zhou did the in vivo animal model.

Figure 3:

Yating Wang did the MitoTracker Red staining. Liping Xiong did the western blot experiments. Jinjin Fan did the confocal image collection.

Figure 4:

Yating Wang did the western blot and Edu analysis.

Figure 5:

Yating Wang and Miaoqing Lu did the experiments of oxygen consumption rate and extracellular acidification rate and mitoSox Red staining. Liping Xiong did the detection of apoptosis analysis.

Figure 6:

Yating Wang did the western blot experiments. Yating Wang and Huiyan Li did the chromatin immunoprecipitation assays and real-time PCR analyses. Miaoqing Lu and Liping Xiong did the immunofluorescence staining.

Signed for and on behalf of the Author(s):

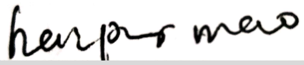

Print Name: Haiping Mao

Date: 2019-12-13
